# Physical activity, polygenic risk score, and colorectal cancer risk

**DOI:** 10.1002/cam4.5072

**Published:** 2022-07-26

**Authors:** Xuechen Chen, Feng Guo, Jenny Chang‐Claude, Michael Hoffmeister, Hermann Brenner

**Affiliations:** ^1^ Division of Clinical Epidemiology and Aging Research German Cancer Research Center (DKFZ) Heidelberg Germany; ^2^ Medical Faculty Heidelberg Heidelberg University Heidelberg Germany; ^3^ Unit of Genetic Epidemiology German Cancer Research Center (DKFZ) Heidelberg Germany; ^4^ Genetic Tumor Epidemiology Group University Medical Center Hamburg‐Eppendorf, University Cancer Center Hamburg Hamburg Germany; ^5^ German Cancer Consortium (DKTK) German Cancer Research Center (DKFZ) Heidelberg Germany; ^6^ Division of Preventive Oncology German Cancer Research Center (DKFZ) and National Center for Tumor Diseases (NCT) Heidelberg Germany

**Keywords:** colorectal cancer, genetic risk equivalent, physical activity, polygenic risk score

## Abstract

**Introduction:**

Whether and to what extent the relationship between physical activity (PA) and colorectal cancer (CRC) differs according to CRC‐related genetic risk remains to be determined, and no studies to date have quantified how much genetically determined risk could be compensated for with active exercise.

**Methods:**

Genetic risk was quantified by a polygenic risk score (PRS) summarizing the estimated effect of 140 CRC‐associated genetic variants. Associations of PA with CRC risk were estimated by multivariable logistic regression across PRS levels. We also compared the impact of PA and specific PA types to the PRS using “genetic risk equivalent (GRE)”, a novel approach to enhance effective risk communication.

**Results:**

Among 5058 CRC patients and 4134 controls, we observed no significant association between overall PA level in quartiles and CRC risk. However, the highest versus lowest lifetime leisure time physical activity (LTPA) was associated with a 13% lower CRC risk [odds ratio 0.87, 95% confidence interval (CI) 0.77–1.00] independent of PRS levels (adjusted *p* value for interaction = 0.18). This effect was equivalent to the effect of having 11 percentiles lower PRS (GRE −10.6, 95% CI −20.7 to −0.6). The GRE (95% CI) for the highest lifetime sports tertile was −23.0 (−33.9 to −12.0).

**Conclusions:**

LTPA was inversely associated with CRC risk irrespective of polygenic risk for CRC, which reinforces the importance of LTPA in CRC prevention among the general population. Adequate sports activity can compensate for a large share of polygenic risk for CRC.

## Graphical abstract

This study, based on a large ongoing case‐control study (the DACHS study) on CRC in Germany, adds to the limited evidence of the individual and joint association of PA and polygenic risk score (PRS) with CRC risk and compares these associations using a novel metric of GRE for effective risk communication. We found that leisure time PA, in particular sports, was associated with reduced CRC risk regardless of PRS levels. The high GREs suggest that adequate sports activity can compensate for a large share of predetermined polygenic risk for CRC.

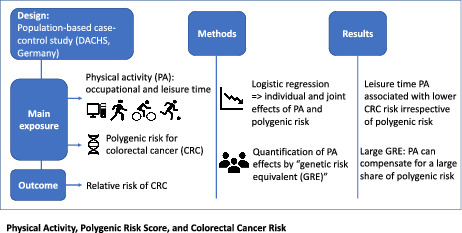

## 1 INTRODUCTION

Despite various potential opportunities for prevention, colorectal cancer (CRC) remains one of the most severe public health issues worldwide, accounting for more than 1.9 million new cases and 0.9 million deaths in 2020.^1^, ^2^ Both environmental and genetic factors contribute to the etiology and progression of CRC.^3^, ^4^, ^5^ Hence, it is important to have a better understanding of their relationship in order to develop targeted prevention strategies.

Substantial evidence has accumulated that adequate physical activity (PA) can serve as an efficient means of preventing CRC development^6^, ^7^, ^8^, ^9^ and improving survival of CRC patients.^10^, ^11^ Studies have tried to explore the interaction between single genetic variants and PA on CRC risk to provide insights into biological mechanisms through which PA might exert its protective effect.^12^, ^13^, ^14^, ^15^, ^16^ Lack of statistically significant interactions in some studies can be partly due to the weak effect of single loci and multiple testing problems.^15^, ^16^ A polygenic risk score (PRS), integrating information across disease‐related genes, may enable more powerful assessment of interactions of PA with genetic predisposition by considering a broad range of genetic susceptibility.^17^, ^18^ Whether and to what extent the association of PA with CRC risk differs by personal genetic backgrounds remains to be determined, and no studies to date have quantified how much higher genetic risk could be compensated for with PA.

Thus, this study was aimed to explore the relationship between PA and CRC risk at different levels of PRS to CRC and to estimate how much increased genetic CRC risk could be compensated for by PA using the recently developed metric “Genetic Risk Equivalent (GRE)”, which may help compare effects of environmental and genetic factors and support effective communication of the potential of prevention.^19^, ^20^

## 2 MATERIALS AND METHODS

### 2.1 Study design and study population

Our analysis is based on data from the DACHS (Darmkrebs: Chancen der Verhütung durch Screening [German]) study initiated in 2003 and carried out in the Rhine‐Neckar region in southwest Germany. Details of the study design have been described elsewhere.^21^, ^22^ Briefly, German‐speaking patients (≥30 years) with the first diagnosis of CRC are recruited from more than 20 hospitals providing CRC surgery in this study region. Controls are randomly drawn from population registries and are frequency matched to cases by age (5‐year age groups), sex, and county of residence. Controls are excluded if they have a history of CRC. The current study was based on cases and controls recruited from 2003 to 2017. During this period, approximately 50% of all eligible cases and 51% of all eligible controls agreed to participate in this study.

Ethical approval for the DACHS study was obtained from the Ethics Committee of the Heidelberg Medical Faculty of Heidelberg University and the state medical boards of Baden‐Wuerttemberg and Rhineland‐Palatinate. Written informed consent was obtained from all participants.

### 2.2 Data collection

Data on sociodemographic characteristics, lifestyles, and medical and family history were collected in an approximately one‐hour personal interview conducted by trained interviewers using a standardized questionnaire. Medical data were extracted for all cases from hospital charts. Interviews for patients were scheduled in hospital during their first hospitalization due to CRC if possible or shortly after discharge and were scheduled at homes for controls. In addition, blood and buccal swab samples were collected. Controls opting out of the interview only provided some key information in a short self‐administered questionnaire, and thus were not included in this analysis.

### 2.3 Assessment of physical activity

Participants were interviewed concerning how much time (hour/week) they spent with hard work, light work, walking, cycling, or doing sports at each decade of life from 20 to 80 years. Participants with missing information from all decades were excluded from our analysis. We calculated specific activity level at each decennial age for each participant according to the task‐specific metabolic equivalent of task (MET) values^23^ (8, 2.5, 3.3, 6, and 8 MET‐hour/week were assumed for each hour per week spent doing hard work, light work, walking, cycling, and sports, respectively). Average lifetime PA was calculated using information from all ages. Information from the most recent decennial age preceding the participants' age was used to derive the latest PA. For example, information from age 50 was used for participants aged 50–59. Data on the activity‐specific MET‐hour/week for walking, cycling, and doing sports were used to create the variables lifetime and latest leisure time physical activity (LTPA).

### 2.4 Derivation of the polygenic risk score

Table S1 provides information about genotyping and imputation of missing genotypes that have been reported elsewhere.^19^, ^24^ The PRS in the current analysis aggregates information from 140 CRC‐related loci identified in a recent genome‐wide association study (Table S2).^4^ It was calculated by summing risk alleles of the respective variants (0, 1, or 2 copies per risk allele for genotyped loci; imputed dosages for imputed loci).^4^

### 2.5 Statistical analysis

Descriptive analyses were used to characterize the distribution of physical activity domains from age 20 to 80 years in the whole population and the distribution of sociodemographic and clinical characteristics of the study population according to disease status.

Then, the association between PA and CRC risk was assessed using multivariable logistic regression. In these analyses, PA and LTPA were categorized according to quartiles among controls. Model 1 was adjusted for the matching factors age and sex. Model 2 was additionally adjusted for school education, body mass index (kg/m^2^, 5–14 years before enrollment), smoking, alcohol consumption, red meat consumption in the previous 12 months, history of colonoscopy, history of diabetes, family history of CRC (history of CRC in a first‐degree relative), current use of statins ≥1 time per week, regular use of non‐steroidal anti‐inflammatory drugs (NSAIDs) ≥2 times per week for more than 1 year, and PRS (continuous variable). Alcohol consumption was categorized according to the recommended maximum limits of 12 and 24 g ethanol daily for women and men, respectively, in Germany.^25^ Detail categories for each variable are presented in Table 1.

**TABLE 1:** Baseline characteristics of the study population

Characteristics	CRC cases	Controls	*p*‐value^a^
	*N* (%)	*N* (%)	
Total	5058	4134	
Sex			
Female	1999 (39.5)	1593 (38.5)	
Male	3059 (60.5)	2541 (61.5)	
Age (year)			
Median (Q1, Q3)	69 (61, 76)	70 (62, 76)	
School education (year)			
<9	3296 (65.2)	2281 (55.2)	
9–10	898 (17.8)	876 (21.2)	<0.0001
>10	855 (16.9)	970 (23.5)	
Average lifetime PA (MET‐hour/week)			
Q1 (≤121.5)	1145 (22.6)	1034 (25.0)	
Q2 (121.6–178.4)	1254 (24.8)	1034 (25.0)	
Q3 (178.5–244.8)	1238 (24.5)	1033 (25.0)	0.0030
Q4 (>244.8)	1421 (28.1)	1033 (25.0)	
Average lifetime LTPA (MET‐hour/week)			
Q1 (≤27.7)	1466 (29.0)	1037 (25.1)	
Q2 (27.8–44.5)	1265 (25.0)	1031 (24.9)	
Q3 (44.6–67.6)	1203 (23.8)	1035 (25.0)	<0.0001
Q4 (>67.6)	1124 (22.2)	1031 (24.9)	
Latest PA (MET‐hour/week)			
Q1 (≤63.2)	1284 (25.4)	1035 (25.0)	
Q2 (63.3–109.5)	1316 (26.0)	1034 (25.0)	
Q3 (109.6–163.9)	1167 (23.1)	1032 (25.0)	0.20
Q4 (>163.9)	1291 (25.5)	1033 (25.0)	
Latest LTPA (MET‐hour/week)			
Q1 (≤15.9)	1514 (29.9)	1061 (25.7)	
Q2 (16.0–33.0)	1435 (28.4)	1025 (24.8)	
Q3 (33.1–61.1)	1062 (21.0)	1016 (24.6)	<0.0001
Q4 (>61.1)	1047 (20.7)	1032 (25.0)	
Smoking status			
Never	2247 (44.4)	2088 (50.5)	
Former	2032 (40.2)	1587 (38.4)	<0.0001
Current	759 (15.0)	448 (10.8)	
Alcohol consumption			
Above recommended threshold	1319 (26.1)	938 (22.7)	<0.001
Red meat intake			
<1 time per week	231 (4.6)	143 (3.5)	
≥1 time per week and <1 time per day	4418 (87.3)	3516 (85.1)	<0.0001
1 time per day	401 (7.9)	472 (11.4)	
BMI (kg/m^2^, 5–14 years before enrollment)			
<25	1526 (30.2)	1573 (38.1)	
25 to <30	2345 (46.4)	1879 (45.5)	<0.0001
30+	1129 (22.3)	650 (15.7)	
History of diabetes	958 (18.9)	559 (13.5)	<0.0001
Family history of colorectal cancer	737 (14.6)	451 (10.9)	<0.0001
Use of NSAIDs	1443 (28.5)	1574 (38.1)	<0.0001
Use of statins	867 (17.1)	929 (22.5)	<0.0001
History of colonoscopy	1342 (26.5)	2495 (60.4)	<0.0001

*Note*: Missing values for cases/controls: school education 9/7, smoking status 20/11, alcohol consumption 11/14, red meat intake 8/3, BMI 58/32, history of diabetes 7/5, family history of colorectal cancer 3/3, use of statins 2/5.

Abbreviations: BMI, body mass index; CRC, colorectal cancer; LTPA, leisure time physical activity; MET, metabolic equivalent of task; NSAID, nonsteroidal anti‐inflammatory drug; PA, physical activity; Q, quartile.

^a^ *p* values were not reported for the matching factors age and sex.

Accounting for potential variations by sex, we tested for the interactions between PA/LTPA or PRS and sex on CRC risk on model 2. The interaction between PRS and PA on CRC risk was tested by including an additional cross‐product term of these variables. P values for interaction analyses were reported with and without adjustment for multiple testing using the FDR (False Discovery Rate) method. Besides, we conducted a stratified analysis by age (<55 years, ≥55 years). We also assessed the individual associations of PRS categories (low, medium, and high levels according to tertiles of PRS among controls) with CRC risk, and estimated odds ratios (ORs) and 95% confidence intervals (CIs) of PA for CRC in participants by PRS categories. Joint associations of PA and PRS with CRC risk were evaluated using participants with a low PRS and the lowest level of PA as reference group.

We then assessed the association between LTPA and CRC risk using the same methods described above. Furthermore, the association of specific types of LTPA (walking, cycling, or sports) with CRC risk was explored. In these analyses, we categorized participants into three groups only, due to large proportions of people not engaging in specific types of LTPA (in particular sports).

### 2.6 Calculation of genetic risk equivalent for PA categories

Details of derivation of GREs (95% CIs) have recently been published elsewhere and are described specifically for this study in Method S1.^19^ The concept of GRE was derived in analogy with the well‐established concept of risk and rate advancement periods.^26^ Here, GREs for PA categories were calculated as ratios of coefficients for PA and PRS percentiles from logistic regression models. Using this approach, we can directly compare effect estimates of PA with effect estimates for increase in PRS by 1 percentile. For example, a GRE of −20 for the effect conveyed by adopting a certain level of physical activity would correspond to an effect that is equivalent to having a 20 percentile lower PRS.

All statistical analyses were carried out using R software, version 4.0.3 (R Foundation for Statistical Computing, Vienna, Austria). Statistically significance was defined as two‐sided *p* values less than 0.05.

## 3 RESULTS

### 3.1 Study population

**FIGURE 1:**
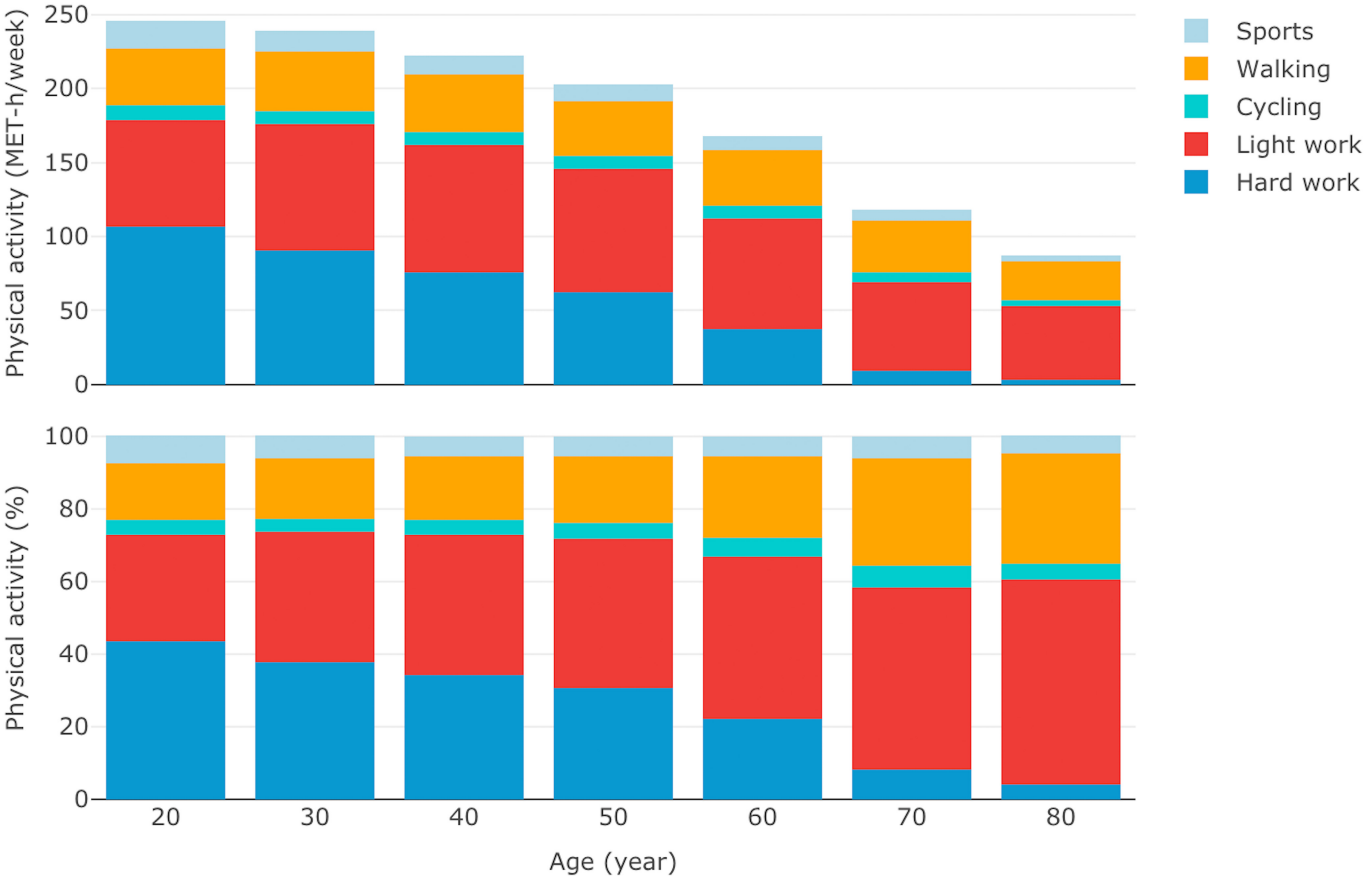
Mean physical activity level from age 20 to 80 years. MET, metabolic equivalent of task.

A total of 5058 CRC cases and 4134 controls were included in this analysis after excluding the participants (66 cases and 14 controls) with missing values of PA information from all decades (Figure S1). Overall PA level decreased from age 20 to 80 years mainly due to the decline in hard work activity level (Figure 1). LTPA level was lower, but the proportion of LTPA to the total level of PA was higher among participants at older ages when compared to the younger ages. 60.5% of cases and 61.5% of controls were men (Table 1). The median age was 69 years for cases and 70 years for controls. Generally, CRC cases tended to be less educated and to have a higher level of average lifetime PA and a lower level of LTPA, and more often drank alcohol or consumed red meat. CRC cases included a higher proportion of current smokers, overweight or obese participants, and participants with a history of diabetes or a family history of CRC than controls. Controls used NSAIDs or statins more often and had more often had a colonoscopy examination before diagnosis.

### 3.2 PA/LTPA and CRC risk

Associations of overall and recreational physical activity or PRS with CRC risk were independent of sex (*p* values for interactions between lifetime PA and LTPA with sex were 0.10 and 0.79, respectively; *p* value for interaction between PRS and sex was 0.90), and therefore, we conducted the association analyses in both sexes. We also did not observe variations of associations between PA and CRC risk by age (Table S3). Although high average lifetime PA was associated with increased CRC risk in age‐ and sex‐adjusted analyses, no significant association between lifetime or latest total PA and CRC risk was observed after adjusting for multiple covariates (Table 2). Further exploratory analyses (results not shown) by adding one covariate at a time into model 1 revealed that the change in the OR of average lifetime PA was mainly driven by adjusting for schooling education, suggesting this factor as the most influential confounder. However, lifetime LTPA was negatively associated with CRC risk. Participants in the fourth quartile of lifetime LTPA levels had a 13% (95% CI, 0%–23%) lower CRC risk, when compared to participants in the first quartile. The interactions of lifetime LTPA with PRS did not reach statistical significance after multiple testing correction (adjusted *p* value for interaction = 0.18).

**TABLE 2:** Association of physical activity with colorectal cancer risk

Variable	CRC cases	Controls	Model 1^a^	Model 2^b^	p‐interaction/*q*‐value^c^
	*N* (%)	*N* (%)	OR (95% CI)	OR (95% CI)	PA/LTPA*PRS
Average lifetime PA					0.53/0.53
Q1	1124 (22.7)	1017 (25.1)	Ref.	Ref.	
Q2	1212 (24.5)	1014 (25.0)	1.08 (0.95, 1.21)	0.99 (0.87, 1.13)	
Q3	1210 (24.5)	1011 (24.9)	1.08 (0.96, 1.21)	1.00 (0.87, 1.14)	
Q4	1395 (28.2)	1014 (25.0)	1.25 (1.11, 1.40)	0.98 (0.85, 1.12)	
p‐trend			<0.001	0.78	
Average lifetime LTPA					0.044/0.18
Q1	1427 (28.9)	1014 (25.0)	Ref.	Ref.	
Q2	1244 (25.2)	1012 (25.0)	0.87 (0.78, 0.98)	0.91 (0.80, 1.03)	
Q3	1171 (23.7)	1014 (25.0)	0.82 (0.73, 0.92)	0.90 (0.79, 1.02)	
Q4	1099 (22.2)	1016 (25.0)	0.76 (0.68, 0.86)	0.87 (0.77, 1.00)	
p‐trend			<0.0001	0.047	
Latest PA					0.23/0.32
Q1	1248 (25.3)	1016 (25.0)	Ref.	Ref.	
Q2	1279 (25.9)	1013 (25.0)	1.02 (0.91, 1.15)	1.09 (0.95, 1.24)	
Q3	1149 (23.3)	1014 (25.0)	0.90 (0.80, 1.02)	1.00 (0.88, 1.15)	
Q4	1265 (25.6)	1013 (25.0)	0.98 (0.86, 1.10)	1.03 (0.90, 1.19)	
p‐trend			0.33	0.90	
Latest LTPA					0.24/0.32
Q1	1470 (29.8)	1035 (25.5)	Ref.	Ref.	
Q2	1403 (28.4)	1011 (24.9)	0.96 (0.86, 1.08)	1.02 (0.90, 1.16)	
Q3	1037 (21.0)	998 (24.6)	0.71 (0.63, 0.80)	0.82 (0.72, 0.94)	
Q4	1031 (20.9)	1012 (25.0)	0.69 (0.61, 0.78)	0.89 (0.78, 1.02)	
p‐trend			<0.0001	0.0084	

Abbreviations: CI, confidence interval; CRC, colorectal cancer; LTPA, leisure time physical activity; OR, odds ratio; PA, physical activity; PRS, polygenic risk score; Q, quartile; Ref., reference.

^a^ Adjusted for age and sex.

^b^ Additionally adjusted for school education, body mass index, smoking status, alcohol consumption, red meat intake, history of colonoscopy, history of diabetes, family history of colorectal cancer, use of statins, use of non‐steroidal anti‐inflammatory drugs, and polygenic risk score (continuous variable).

^c^ Tested by additionally including a cross‐term of PRS and physical activity in model 2; The *q*‐values are the FDR‐adjusted *p* values.

**FIGURE 2:**
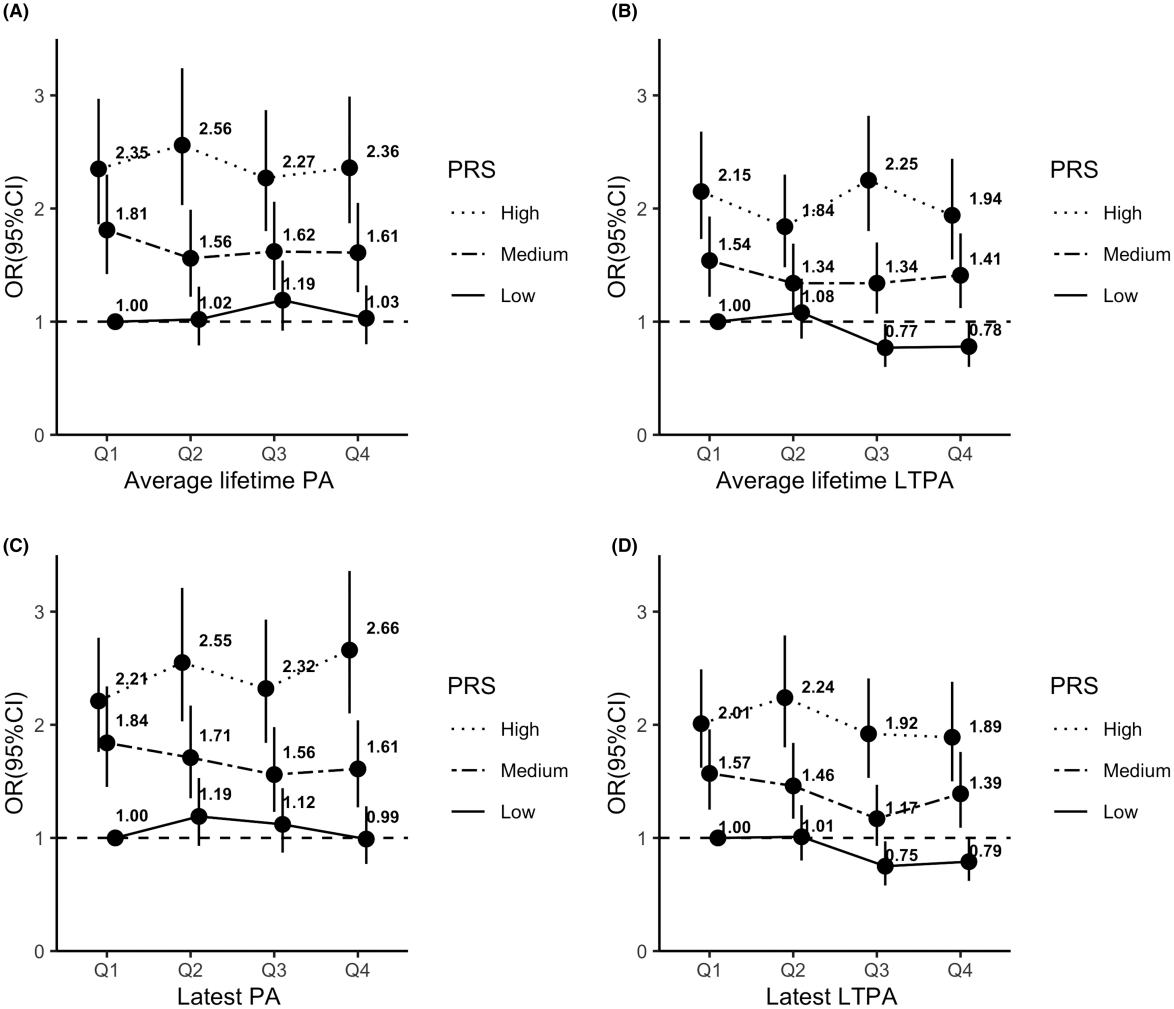
Association of physical activity with colorectal cancer risk by polygenic risk score. *Note*: ORs (95% CIs) were estimated with participants in the lowest PA (Q1) and with low PRS as reference; PRS was categorized into three groups (low, medium, and high PRS) according to the tertiles of PRS among controls; Models were adjusted for age, sex, school education, body mass index, smoking, alcohol consumption, red meat consumption, history of colonoscopy, history of diabetes, family history of colorectal cancer, use of statins, and use of non‐steroidal anti‐inflammatory drugs. CI, confidence interval; LTPA, leisure time physical activity; OR, odds ratio; PA, physical activity; PRS, polygenic risk score; Q, quartile.

Participants who had a high or medium level of PRS had a 2.3‐ or 1.6‐fold increased risk of CRC, respectively, when compared to those with a low PRS level (Table 3 **)**. The OR (95% CI) for per 10 percentiles increase in PRS was 1.14 (1.12, 1.16). Participants with the highest level of lifetime LTPA had a GRE of −10.6 (95% CI, −20.7 to 0.6), which can be interpreted that high lifetime LTPA could compensate for about 11 percentiles of less favorable genetic predisposition to CRC. Similar results were obtained for the latest LTPA. In addition, ORs showed similar patterns of variation across different categories of PA for people with low, medium, and high levels of PRS (Table S4 and Figure 2).

**TABLE 3:** Genetic risk equivalent for comparisons between physical activity categories

Variable	OR (95% CI)^a^	GRE (95% CI)	Variable	OR (95% CI)^a^	GRE (95% CI)
PRS			PRS		
Low	Ref.		Low	Ref.	
Medium	1.56 (1.39, 1.76)		Medium	1.56 (1.39, 1.76)	
High	2.26 (2.02, 2.54)		High	2.26 (2.02, 2.54)	
per 10 percentiles	1.14 (1.12, 1.16)		per 10 percentiles	1.14 (1.12, 1.16)	
Average lifetime PA			Latest PA		
Q1	Ref.	Ref.	Q1	Ref.	Ref.
Q2	0.99 (0.87, 1.13)	−0.8 (−10.9, 9.4)	Q2	1.08 (0.95, 1.23)	5.9 (−4.1, 15.9)
Q3	1.00 (0.87, 1.14)	0 (−10.3, 10.3)	Q3	1.00 (0.87, 1.14)	0 (−10.3, 10.3)
Q4	0.98 (0.85, 1.12)	−1.5 (−12.0, 9.0)	Q4	1.03 (0.90, 1.19)	2.3 (−8.3, 12.8)
PRS			PRS		
Low	Ref.		Low	Ref.	
Medium	1.56 (1.39, 1.76)		Medium	1.57 (1.39, 1.77)	
High	2.26 (2.02, 2.54)		High	2.27 (2.02, 2.54)	
per 10 percentiles	1.14 (1.12, 1.16)		per 10 percentiles	1.14 (1.12, 1.16)	
Average lifetime LTPA			Latest LTPA		
Q1	Ref.	Ref.	Q1	Ref.	Ref.
Q2	0.91 (0.80, 1.03)	−7.2 (−17.0, 2.6)	Q2	1.02 (0.90, 1.16)	1.5 (−8.1, 11.1)
Q3	0.90 (0.79, 1.02)	−8.0 (−18.0, 1.9)	Q3	0.82 (0.72, 0.94)	−15.1 (−25.5, −4.8)
Q4	0.87 (0.77, 0.99)	−10.6 (−20.7, −0.6)	Q4	0.88 (0.77, 1.01)	−9.8 (−20.1, 0.6)

Abbreviations: CI, confidence interval; GRE, genetic risk equivalent; LTPA, leisure time physical activity; OR, odds ratio; PA, physical activity; PRS, polygenic risk score; Q, quartile; Ref., reference.

^a^ Variables in models included age, sex, school education, body mass index, smoking status, alcohol consumption, red meat intake, history of colonoscopy, history of diabetes, family history of colorectal cancer, use of statins, use of non‐steroidal anti‐inflammatory drugs, PA/LTPA, and PRS (per 10 percentiles, continuous variable, for the analysis of PA/LTPA)/PRS (categorical variable, categorized according to tertiles of PRS among controls).

### 3.3 Specific LTPA and CRC risk

Table 4 shows the associations between specific LTPA and CRC risk. Cases tended to engage in less LTPA during their lifetime when compared to controls. No significant associations were observed between lifetime cycling or walking and CRC risk, and results for trend analyses did not reach statistical significance. A high level of lifetime sports was associated with a strong risk reduction of CRC by 26% (95% CI, 16%–36%) when compared to those with a low level. The corresponding GRE was −23 (95% CI, −34 to −12). Similar activity‐specific associations with CRC risk were seen for the latest LTPA.

**TABLE 4:** Association of specific physical activity with colorectal cancer risk

Variable	CRC cases	Controls	OR (95% CI)^a^	GRE	Variable	CRC cases	Controls	OR (95% CI)^a^	GRE
	*N* (%)	*N* (%)		(95% CI)		*N* (%)	*N* (%)		(95% CI)
PRS					PRS				
Low	1006 (21.0)	1303 (33.3)	Ref.		Low	1006 (21.0)	1303 (33.3)	Ref.	
Medium	1539 (32.1)	1310 (33.5)	1.55 (1.37, 1.75)		Medium	1539 (32.1)	1310 (33.5)	1.55 (1.37, 1.75)	
High	2248 (46.9)	1295 (33.1)	2.26 (2.01, 2.54)		High	2248 (46.9)	1295 (33.1)	2.26 (2.01, 2.54)	
per 10 percentiles			1.14 (1.12, 1.16)		per 10 percentiles			1.14 (1.12, 1.16)	
Average lifetime cycling^b^					Latest cycling^b^				
Low	1589 (33.2)	1299 (33.2)	Ref.	Ref.	Low	2402 (50.1)	1779 (45.5)	Ref.	Ref.
Medium	1600 (33.4)	1303 (33.3)	1.09 (0.97, 1.22)	6.6 (−2.3, 15.5)	Medium	1057 (22.1)	923 (23.6)	0.93 (0.83, 1.05)	−5.5 (−14.9, 3.8)
High	1604 (33.5)	1306 (33.4)	1.12 (0.98, 1.28)	8.6 (−1.7, 19.0)	High	1334 (27.8)	1206 (30.9)	0.94 (0.83, 1.07)	−4.7 (−14.8, 5.3)
p‐trend			0.093		p‐trend			0.30	
Average lifetime walking^b^					Latest walking^b^				
Low	1757 (36.7)	1303 (33.3)	Ref.	Ref.	Low	2049 (42.7)	1547 (39.6)	Ref.	Ref.
Medium	1538 (32.1)	1304 (33.4)	0.92 (0.82, 1.03)	−6.4 (−15.3, 2.6)	Medium	1559 (32.5)	1298 (33.2)	0.96 (0.86, 1.08)	−3.1 (−11.7, 5.4)
High	1498 (31.3)	1301 (33.3)	0.90 (0.79, 1.03)	−8.0 (−18.2, 2.1)	High	1185 (24.7)	1063 (27.2)	0.99 (0.86, 1.13)	−0.8 (−11.2, 9.7)
p‐trend			0.12		p‐trend			0.79	
Average lifetime sports^b^					Latest sports^b^				
Low	2080 (43.4)	1307 (33.4)	Ref.	Ref.	Low	3425 (71.5)	2369 (60.6)	Ref.	Ref.
Medium	1440 (30.0)	1348 (34.5)	0.75 (0.67, 0.84)	−22.0 (−31.0, −12.9)	Medium	373 (7.8)	409 (10.5)	0.74 (0.63, 0.88)	−23.0 (−36.0, −10.0)
High	1273 (26.6)	1253 (32.1)	0.74 (0.64, 0.84)	−23.0 (−33.9, −12.0)	High	995 (20.8)	1130 (28.9)	0.73 (0.64, 0.83)	−24.0 (−34.3, −13.8)
p‐trend			<0.0001		p‐trend			<0.0001	

Abbreviations: CI, confidence interval; CRC, colorectal cancer; GRE, genetic risk equivalent; OR, odds ratio; PRS, polygenic risk score; Ref., reference.

^a^ Variables in the models included age, sex, school education, body mass index, smoking status, alcohol consumption, red meat intake, history of colonoscopy, history of diabetes, family history of colorectal cancer, use of statins, use of non‐steroidal anti‐inflammatory drugs, specific LTPA, LTPA (for the analysis of lifetime cycling/walking/sports), and latest LTPA (for the analysis of latest cycling/walking/sports), and PRS (per 10 percentiles, continuous variable, for the analysis of specific LTPA)/PRS (categorical variable, categorized according to tertiles of PRS among controls).

^b^ Categorized into three groups according to tertiles of respective physical activity levels among controls.

## 4 DISCUSSION

Higher leisure time activity was associated with reduced CRC risk, and the association was most pronounced for sports activity in our study. These associations were independent of PRS levels, which underscore the importance of promoting active LTPA in the general population regardless of their predetermined polygenic risk for CRC. The high GRE for sports activity implies that a large share of predetermined genetic risk can be compensated for by vigorous‐intensity PA.

Consistent with previous studies^6^, ^8^, ^9^ underscoring the difference in the association of PA domains (such as occupational domain, recreational domain, and household domain) with CRC risk, our study shows that LTPA was associated with reduced CRC risk. Participants with average lifetime LTPA in the fourth quartile (>67.6 MET‐hours/week) had a 13% lower CRC risk when compared to the group in the first quartile in this study, which is in line with the finding from the World Cancer Research Fund Network in 2018 (highest versus lowest: pooled relative risk 0.84, 95% CI 0.78, 0.91).^27^ LTPA levels of most participants in our study were higher than the World Health Organization (WHO) recommended PA level (10 MET‐hours/week^28^) to reduce non‐communicable diseases. According to a recent dose–response meta‐analysis by Liu et al,^7^ reduction of CRC risk dropped dramatically with less than 20 MET‐hours/week, and only 7% risk reduction of CRC could be observed with the relatively low level recommended by WHO. In line with previous studies, our findings, therefore, indicate that a more substantial reduction of CRC risk might be possible with LTPA levels well above those recommended by WHO.

Although adequate moderate‐ or vigorous‐intensity PA is recommended to improve public health,^28^ there is still a lack of evidence based on which to recommend specific PA, in particular the dose of specific types of PA. We observed a stronger association of CRC risk with the total volume of sports activity (>6 METs) than with cycling or walking. These most important types of LTPA were specifically addressed in our study to facilitate translation of results in potential implications for prevention. Several commonly hypothesized mechanistic pathways of exercise effects (sex hormones, metabolic hormones, immune function, et al) have been proposed,^29^ while the effects and effect size might vary depending on training intensity.^30^, ^31^, ^32^ For example, vigorous‐intensity exercise might have an additional positive effect by producing an antioxidant response in tumors that will result in an antiproliferative effect, which mainly depends on the chosen modality and intensity of training.^32^ Our results support suggestions that type‐specific effects of LTPA should be taken into consideration when analyzing the effects of LTPA on CRC, given that recreational activity varies in terms of its frequency, intensity, and physiological effects.

Recent studies have shown that healthy lifestyles including PA are associated with a substantially reduced risk of CRC regardless of individuals' genetic risk.^33^, ^34^ However, evidence on the joint relationship of single components of healthy lifestyles like PA and integrative measures of genetic risk such as PRS with CRC risk has remained limited. A recent study by Yang et al examined the interaction between overall PA level and PRS in the UK Biobank and a case–control study of CRC from Scotland, but they did not find any significant results.^35^ We likewise did not observe a significant interaction between PRS and overall PA or for any of the specific types of LTPA, despite the very large sample size of our study. Both the study by Yang et al and our study examined multiplicative interactions that can be directly estimated from logistic regressions and are more suitable for causal assessment.^36^ It should be noted, however, that the absence of interaction on the multiplicative scale does not imply the absence of interaction with respect to absolute risk. In fact, comparable relative risk at various levels of PRS implies that absolute risk reduction by LTPA would even be higher and most important for those in the highest genetic risk group. Nevertheless, it is important to note that our results also point to major beneficial effects of LTPA for those with low PRS. They therefore should not be misinterpreted as LTPA being relevant for those at high PRS only.

The interplay between lifestyle and genetic factors in the development of CRC has resulted in a complex risk communication, an important aspect in cancer prevention. A novel component of this study is that we applied our recently developed metric GRE to directly compare the effect estimates of PA to the effect estimates of PRS. This metric might be easier to comprehend and more useful for risk communication when compared to the traditional metrics like odds ratios. The large GRE implies that a substantial share of background polygenic risk for CRC could be compensated for with reduction of risk factors or preventive measures, which might be helpful to communicate risk estimates and the potential for prevention.

Major strengths of the current study include the large sample size and detailed data of the DACHS study, making it possible to investigate the relationship between different PA measures and CRC risk with thorough adjustments. Furthermore, this study for the first time comprehensively explores the interaction of PA/LTPA with the predetermined polygenic risk for CRC and compares their effects using the novel approach of GRE recently developed to enhance effective risk communication.

There are also some limitations that merit attention. First, we cannot draw any causal conclusions because of the observational study design. Second, like in most other epidemiological studies, all PA measures were derived from self‐reports yielding the potential for recall or other information bias. For example, it might be quite hard for elder participants to recall their PA levels at early ages and the recalled PA levels might be related to their current interests, which could have led to misclassification. Use of objective methods to measure PA might reduce bias and measurement error but is limited by cost, time, and complexity of data processing in a large population‐based sample and essentially unfeasible for retrospective collections of relevant lifetime and recent exposure information in case–control studies. Third, despite a most detailed ascertainment of various types of PA throughout adulthood, further potentially relevant domains of PA, such as household activity, were not included in our study,^23^ which partly limits comparability of our results with those of other studies that had specifically focused on such domains.^8^, ^9^ Fourth, residual confounding by less than perfect and incomplete information cannot be excluded. For example, although we have adjusted for multiple factors that are associated with CRC risk and also PA levels, such as diabetes, there may be other health conditions such as respiratory health that might have affected both PA and CRC risk. Fifth, all analyses were based on a Caucasian population, thus more research on other ethnic groups is warranted to validate our results.

In conclusion, our study suggests that LTPA, in particular sports, may make a major contribution in preventing CRC. Absence of interaction with PRS on the multiplicative scale suggests a similar inverse association with CRC at different levels of genetic risk, although absolute risk reduction would be expected to be more pronounced for those with a high PRS. The high GREs reinforce the importance of LTPA, especially sports activity, in CRC prevention and may be helpful in communicating this important message to the general population. Further research is warranted to more precisely estimate the preventive potential of specific types of PA and to clarify the mechanism behind the observed associations.

## AUTHOR CONTRIBUTION

Conceptualization and design: H.B.; Acquisition of data: J.C.‐C., M.H., H.B.; Analysis and interpretation of data: X.C., F.G., M.H., H.B.; Writing‐original draft: X.C., H.B.; Writing‐review & editing: all authors; Study supervision: J.C.‐C., M.H., H.B. All authors read and approved the final manuscript.

## FUNDING INFORMATION

The DACHS study was funded by the German Research Council (BR 1704/6–1, BR1704/6–3, BR 1704/6–4, BR 1704/6–6, CH 117/1–1, BR 1704/17–1, HO 5117/2–1) and the German Federal Ministry of Education and Research (01KH0404, 01ER0814, 01ER0815, 01GL1712). The sponsors had no role in the study design, in the collection, analysis, and interpretation of data and preparation, review or approval of the manuscript.

## CONFLICT OF INTEREST

The authors declare no conflict of interest.

## ETHICS APPROVAL

Ethical approval for the DACHS study was obtained from the Ethics Committee of the Heidelberg Medical Faculty of Heidelberg University and the state medical boards of Baden‐Wuerttemberg and Rhineland‐Palatinate.

## Supporting information


Appendix S1

## Data Availability

Data are available from the corresponding author upon reasonable request.
