# Supplementary material for: Physical activity, polygenic risk score, and colorectal cancer risk
Source: Cancer Med. 2022 Jul 26;12(4):4655–66. doi: 10.1002/cam4.5072 (PMC9972112; doi:10.1002/cam4.5072)
Supplement: Supplementary file 1 — Appendix S1 [file CAM4-12-4655-s001.docx]

**Supplementary Method**

***Derivation of genetic risk equivalent (GRE)***

The concept of GRE was in analogy with the well-established concept of risk and rate advancement periods ^1^. Briefly, consider an analysis based on a multivariable logistic regression:

$$\ln\left( R \right)=a + b1\boldsymbol{\cdot}A + b2\boldsymbol{\cdot}P + \sum_{i=1}^{n} ci\cdot Fi$$

where ln(R) reflects the log odds of the disease risk, and a, b_1_, b_2_ and c_i_ (i = 1, . . ., n) refer to the intercept and model parameters for A [physical activity (PA), categorized as 1 for group with active PA and 0 for the reference group with lowest level of PA], P (PRS percentiles according to the distribution of PRS among controls), and F (other covariates), respectively. The GRE was calculated as the ratio of b_1_ and b_2,_ the estimated coefficients for PA categories and the PRS from the regression models, and thus the properties of GRE follow from the properties of b_1_ and b_2_, which include consistency, asymptotic unbiasedness, and normality. Using the delta method ^2^, the asymptotic variance of GRE can be derived as:

$$\mathrm{var}\left( \mathrm{GRE} \right)=\frac{1}{{b_{2}}^{2}}\left[ \mathrm{var}\left( b_{1} \right)-2.\left( \frac{b_{1}}{b_{2}} \right).cov\left( b_{1},b_{2} \right)+\left( \frac{b_{1}}{b_{2}} \right)^{2}.var\left( b_{2} \right) \right]$$

As the GRE is asymptotically normal, its 95% confidence intervals can be easily calculated using the square root of var(GRE) and sample size (n):

$$GRE\pm1.96\sqrt{\frac{var(GRE)}{n}}$$

^1^Brenner H, Gefeller O, Greenland S. Risk and rate advancement periods as measures of exposure impact on the occurrence of chronic diseases. Epidemiology 1993; **4**:229–36.

^2^**Bishop** YMM, **Fienberg** SE, **Holland** PW. Discrete Multivariate Analysis: Theory and Practice. Cambridge, MA; MIT press, 1975.

**Supplementary Table S1**. Information about genotyping and imputation

| **Genotyping platform** | **Cases (N)** | **Controls (N)** | **Recruitment Period** | **Imputation** |
| --- | --- | --- | --- | --- |
| Illumina HumanCytoSNP | 1667 | 1699 | 2003-2008 | Haplotype Reference Consortium (version r1.1.2016) |
| Illumina HumanOmniExpress | 664 | 498 | 2007-2010 |  |
| Illumina HumanOmniExpress | 1180 | 626 | 2010-2015 |  |
| Illumina Infinium OncoArray | 894 | 657 | 2003-2016 |  |
| Illumina Global Screening Array | 653 | 654 | 2016-2017 |  |

**NOTE:** We excluded triallelic SNPs, genotyped SNPs which had a low call rate (<98%), lack of Hardy-Weinberg equilibrium in control individuals (p<1×10^–4^), or low minor allele frequency (<0.1%), and those not assigned an rs number. More details can be found in the previous studies by Peters et al^a^ and Schumacher et al^b^.

^a^Peters U, Jiao S, Schumacher FR, et al. Identification of genetic susceptibility loci for colorectal tumors in a genome-wide meta-analysis. Gastroenterology 2013; **144**:799–807.

^b^Schumacher FR, Schmit SL, Jiao S, et al. Genome-wide association study of colorectal cancer identifies six new susceptibility loci. Nat Commun 2015; **6**:7138.

**Supplementary Table S2.** Overview on colorectal cancer related single-nucleotide polymorphisms that were identified in genome-wide association studies and considered in this analysis

| **SNP** | **Locus** | **Position** | **Risk allele** | **Beta** |
| --- | --- | --- | --- | --- |
| rs4360494 | 1p34.3 | 38455891 | G | 0.0379 |
| rs12144319 | 1p32.3 | 55246035 | C | 0.0661 |
| rs72647484 | 1p36.12 | 22587728 | T | 0.0504 |
| rs7542665 | 1p31.3 | 62673037 | C | 0.0334 |
| rs6678517 | 1q25.3 | 183002639 | A | 0.073 |
| rs17011141 | 1q41 | 222112634 | G | 0.0877 |
| rs448513 | 2q24.2 | 159964552 | C | 0.0054 |
| rs11884596 | 2q33.1 | 199612407 | C | 0.0342 |
| rs983402 | 2q33.1 | 199781586 | T | 0.0622 |
| rs7606562 | 2p16.3 | 48686695 | T | 0.0414 |
| rs11692435 | 2q11.2 | 98275354 | G | 0.0492 |
| rs3731861 | 2q35 | 219191256 | T | 0.0613 |
| rs10049390 | 3q22.2 | 133701119 | A | 0.0455 |
| rs13086367 | 3q13.2 | 112903888 | A | 0.0463 |
| rs72942485 | 3q13.2 | 112999560 | G | 0.0545 |
| rs9831861 | 3p21.1 | 53088285 | G | 0.0294 |
| rs35470271 | 3p22.1 | 40915239 | G | 0.0994 |
| rs12635946 | 3q13.2 | 112916918 | C | 0.0334 |
| rs113569514 | 3q22.2 | 133748789 | T | 0.0414 |
| rs9876206 | 3q26.2 | 169517436 | C | 0.0453 |
| rs6781752 | 3p14.1 | 66365163 | A | 0.0597 |
| rs11727676 | 4q31.21 | 145659064 | C | 0.0093 |
| rs1391441 | 4q24 | 106128760 | A | 0.0148 |
| rs13149359 | 4q22.2 | 94938618 | A | 0.052 |
| rs7708610 | 5p13.1 | 40102443 | A | 0.0384 |
| rs78368589 | 5p15.33 | 1240204 | T | 0.0786 |
| rs145364999 | 5q21.1 | 98206082 | T | 0.3496 |
| rs2735940 | 5p15.33 | 1296486 | G | 0.0865 |
| rs12514517 | 5p13.1 | 40280076 | A | 0.1013 |
| rs755229494 | 5q22.2 | 112097351 | G | 0.6286 |
| rs12659017 | 5q23.2 | 125988175 | G | 0.0374 |
| rs4976270 | 5q31.1 | 134467220 | C | 0.0693 |
| rs13204733 | 6p12.1 | 55566108 | G | 0.0643 |
| rs116685461 | 6p21.33 | 31315512 | G | 0.0655 |
| rs9271695 | 6p21.32 | 32593080 | G | 0.0889 |
| rs2516420 | 6p21.33 | 31449620 | C | 0.1091 |
| rs116353863 | 6p21.33 | 31010185 | C | 0.1202 |
| rs16878812 | 6p21.31 | 35569562 | A | 0.0778 |
| rs9470361 | 6p21.2 | 36623379 | A | 0.054 |
| rs62404966 | 6p12.1 | 55712124 | C | 0.0724 |
| rs3131043 | 6p21.33 | 30758466 | G | 0.0294 |
| rs2070699 | 6p24.1 | 12292772 | T | 0.0294 |
| rs1476570 | 6p22.1 | 29809860 | A | 0.0492 |
| rs3830041 | 6p21.32 | 32191339 | T | 0.0645 |
| rs6928864^a^ | 6q21 | 105966894 | C | 0.0531 |
| rs62396735 | 6p21.1 | 41702582 | C | 0.033 |
| rs12672022 | 7p13 | 45136423 | T | 0.0067 |
| rs80077929 | 7p12.3 | 46094089 | T | 0.0093 |
| rs10951878 | 7p12.3 | 46926695 | C | 0.0531 |
| rs3801081 | 7p12.3 | 47511161 | G | 0.0253 |
| rs7013278 | 8q24.21 | 128414892 | T | 0.0091 |
| rs4313119 | 8q24.21 | 128571855 | G | 0.0518 |
| rs16892766 | 8q23.3 | 117630683 | C | 0.2099 |
| rs6469654 | 8q23.3 | 117632965 | G | 0.0677 |
| rs117079142 | 8q24.11 | 117790914 | A | 0.1139 |
| rs6983267 | 8q24.21 | 128413305 | G | 0.1052 |
| rs34405347 | 9q22.33 | 101679752 | T | 0.0089 |
| rs1537372 | 9p21.3 | 22103183 | G | 0.012 |
| rs10980628 | 9q31.3 | 113671403 | C | 0.0511 |
| rs12217641 | 10p14 | 8663875 | C | 0.0069 |
| rs10786560 | 10q24.2 | 101315166 | G | 0.0082 |
| rs1250567 | 10q22.3 | 81046265 | C | 0.047 |
| rs11255841 | 10p14 | 8739580 | T | 0.1064 |
| rs10821907 | 10q11.23 | 52648454 | C | 0.073 |
| rs704017 | 10q22.3 | 80819132 | G | 0.0765 |
| rs11190164 | 10q24.2 | 101351704 | G | 0.0889 |
| rs12246635 | 10q25.2 | 114288619 | C | 0.0975 |
| rs11196170 | 10q25.2 | 114722621 | A | 0.0527 |
| rs7946853 | 11q13.4 | 74409077 | C | 0.0119 |
| rs55864876 | 11q22.1 | 100717136 | G | 0.015 |
| rs2186607 | 11q22.1 | 101656397 | T | 0.0483 |
| rs61389091 | 11q13.4 | 74427921 | C | 0.1934 |
| rs4450168 | 11p15.4 | 10286755 | C | 0.0413 |
| rs174533 | 11q12.2 | 61549025 | G | 0.0636 |
| rs7121958 | 11q13.4 | 74280012 | G | 0.078 |
| rs3087967 | 11q23.1 | 111156836 | T | 0.1122 |
| rs4759277 | 12q13.3 | 57533690 | A | 0.0285 |
| rs1427760 | 12q24.21 | 115100714 | C | 0.0424 |
| rs3217874 | 12p13.32 | 4400808 | T | 0.0453 |
| rs10849433 | 12p13.31 | 6406904 | C | 0.0468 |
| rs11610543 | 12q12 | 43134191 | G | 0.0474 |
| rs35808169 | 12p13.32 | 4368607 | C | 0.089 |
| rs3217810 | 12p13.32 | 4388271 | T | 0.1181 |
| rs2250430 | 12p13.31 | 6421174 | T | 0.0597 |
| rs77969132 | 12p11.21 | 31594813 | T | 0.1583 |
| rs12372718 | 12q13.12 | 51171090 | G | 0.0896 |
| rs597808 | 12q24.12 | 111973358 | G | 0.0737 |
| rs7300312 | 12q24.21 | 115890922 | C | 0.066 |
| rs2710310 | 12p13.2 | 12035649 | C | 0.0145 |
| rs78341008 | 13q22.1 | 73791554 | C | 0.0109 |
| rs8000189 | 13q34 | 111075881 | T | 0.0473 |
| rs45597035 | 13q22.1 | 73649152 | A | 0.0495 |
| rs1924816 | 13q22.1 | 73997961 | A | 0.0506 |
| rs7333607 | 13q13.3 | 37462010 | G | 0.0758 |
| rs1330889 | 13q22.3 | 78609615 | C | 0.0453 |
| rs377429877 | 13q13.2 | 34092164 | C | 0.0468 |
| rs1951864 | 14q22.2 | 54369299 | A | 0.0059 |
| rs17094983 | 14q23.1 | 59189361 | G | 0.0062 |
| rs8020436 | 14q23.1 | 59208437 | A | 0.0294 |
| rs35107139 | 14q22.2 | 54419106 | C | 0.0912 |
| rs4901473 | 14q22.2 | 54445157 | G | 0.0465 |
| rs745213 | 15q23 | 68060389 | G | 0.0072 |
| rs12594720 | 15q22.31 | 67007018 | C | 0.0246 |
| rs56324967 | 15q22.33 | 67402824 | C | 0.0689 |
| rs17816465 | 15q13.3 | 33156386 | A | 0.069 |
| rs12708491 | 15q13.3 | 32992836 | G | 0.0464 |
| rs2293581 | 15q13.3 | 33010736 | A | 0.1248 |
| rs7495132 | 15q26.1 | 91172901 | T | 0.0453 |
| rs9930005 | 16q23.2 | 80043258 | C | 0.0061 |
| rs12447408 | 16q24.1 | 86252544 | A | 0.0079 |
| rs9924886 | 16q22.1 | 68743939 | A | 0.055 |
| rs12149163 | 16q24.1 | 86339315 | T | 0.0487 |
| rs62042090 | 16q24.1 | 86703949 | T | 0.0481 |
| rs983318 | 17q24.3 | 70413253 | A | 0.0397 |
| rs73975586 | 17p13.3 | 814243 | A | 0.0497 |
| rs1078643 | 17p12 | 10707241 | A | 0.0747 |
| rs75954926 | 17q25.3 | 81061048 | G | 0.0882 |
| rs373585858 | 17q25.3 | 80394556 | A | 0.1103 |
| rs4968127 | 17p13.3 | 809643 | G | 0.0514 |
| rs11874392 | 18q21.1 | 46453156 | A | 0.1606 |
| rs73068325 | 19q13.43 | 59079096 | T | 0.0066 |
| rs34797592 | 19p13.11 | 16417198 | T | 0.0824 |
| rs28840750 | 19q13.11 | 33519927 | T | 0.1939 |
| rs1963413 | 19q13.2 | 41871573 | A | 0.0441 |
| rs12979278 | 19q13.33 | 49218602 | T | 0.0293 |
| rs2738783 | 20q13.33 | 62308612 | T | 0.006 |
| rs6067417 | 20q13.13 | 48983697 | C | 0.0331 |
| rs6031311 | 20q13.12 | 42666475 | T | 0.0362 |
| rs6091189 | 20q13.13 | 49256285 | T | 0.0549 |
| rs994308 | 20p12.3 | 6603622 | C | 0.0626 |
| rs28488 | 20p12.3 | 6762221 | T | 0.0714 |
| rs556532366 | 20p12.3 | 8568071 | T | 0.0715 |
| rs189583 | 20p12.3 | 6376457 | G | 0.0795 |
| rs4813802 | 20p12.3 | 6699595 | G | 0.0819 |
| rs11087784 | 20p12.3 | 7740976 | G | 0.0874 |
| rs6066825 | 20q13.13 | 47340117 | A | 0.0719 |
| rs6063514 | 20q13.13 | 49055318 | C | 0.0547 |
| rs13831 | 20q13.32 | 57475191 | G | 0.0334 |
| rs1741640 | 20q13.33 | 60932414 | C | 0.1146 |
| rs6058093 | 20q11.22 | 33213196 | C | 0.045 |

**Abbreviation:** A, adenine; C, cytosine; G, guanine; OR, odds ratio; T, thymine; SNP, single-nucleotide polymorphism.

^a^For building the PRS, the missing reference SNP was replaced by rs6904092 (linkage disequilibrium, D’=1 and r^2^=1).

**Supplementary Table S3.** Association of physical activity with colorectal cancer risk by age

|  | **Age <55 years (N=974)** | | |  | **Age ≥55 years (N=8023)** | | |
| --- | --- | --- | --- | --- | --- | --- | --- |
| **Variable** | **CRC Cases** | **Controls** | **OR (95% CI)^a^** |  | **CRC Cases** | **Controls** | **OR (95% CI)^a^** |
|  | **N (%)** | **N (%)** |  |  | **N (%)** | **N (%)** |  |
| Average lifetime PA | | |  |  |  |  |  |
| Q1 | 123 (21.8) | 103 (25.1) | Ref. |  | 1001 (22.9) | 914 (25.1) | Ref. |
| Q2 | 124 (22.0) | 118 (28.7) | 0.82 (0.55, 1.21) |  | 1088 (24.9) | 896 (24.6) | 1.02 (0.88, 1.17) |
| Q3 | 144 (25.6) | 98 (23.8) | 1.11 (0.74, 1.66) |  | 1066 (24.3) | 913 (25.0) | 0.99 (0.86, 1.15) |
| Q4 | 172 (30.6) | 92 (22.4) | 1.16 (0.76, 1.75) |  | 1223 (27.9) | 922 (25.3) | 0.96 (0.83, 1.12) |
| p-interaction with age^b^ =0.16 | | | | | | | |
|  |  |  |  |  |  |  |  |
| Average lifetime LTPA | | |  |  |  |  |  |
| Q1 | 129 (22.9) | 83 (20.2) | Ref. |  | 1298 (29.6) | 931 (25.5) | Ref. |
| Q2 | 126 (22.4) | 110 (26.8) | 0.70 (0.46, 1.05) |  | 1118 (25.5) | 902 (24.7) | 0.94 (0.82, 1.08) |
| Q3 | 152 (27.0) | 102 (24.8) | 1.07 (0.71, 1.62) |  | 1019 (23.3) | 912 (25.0) | 0.88 (0.77, 1.01) |
| Q4 | 156 (27.7) | 116 (28.2) | 0.92 (0.61, 1.37) |  | 943 (21.5) | 900 (24.7) | 0.88 (0.76, 1.01) |
| p-interaction with age^b^ =0.12 | | | |  |  |  |  |
|  |  |  |  |  |  |  |  |
| Latest PA | |  |  |  |  |  |  |
| Q1 | 77 (13.7) | 56 (13.6) | Ref. |  | 1171 (26.7) | 960 (26.3) | Ref. |
| Q2 | 75 (13.3) | 64 (15.6) | 0.86 (0.51, 1.45) |  | 1204 (27.5) | 949 (26.0) | 1.11 (0.97, 1.27) |
| Q3 | 142 (25.2) | 109 (26.5) | 0.97 (0.60, 1.54) |  | 1007 (23.0) | 905 (24.8) | 1.02 (0.89, 1.18) |
| Q4 | 269 (47.8) | 182 (44.3) | 0.97 (0.63, 1.51) |  | 996 (22.8) | 831 (22.8) | 1.06 (0.91, 1.23) |
| p-interaction with age^b^ =0.64 | | | |  |  |  |  |
|  |  |  |  |  |  |  |  |
| Latest LTPA | |  |  |  |  |  |  |
| Q1 | 111 (19.7) | 75 (18.2) | Ref. |  | 1359 (31.0) | 960 (26.3) | Ref. |
| Q2 | 151 (26.8) | 90 (21.9) | 1.10 (0.72, 1.70) |  | 1252 (28.6) | 921 (25.3) | 1.02 (0.89, 1.16) |
| Q3 | 151 (26.8) | 132 (32.1) | 0.91 (0.60, 1.37) |  | 886 (20.2) | 866 (23.8) | 0.83 (0.72, 0.95) |
| Q4 | 150 (26.6) | 114 (27.7) | 0.95 (0.62, 1.44) |  | 881 (20.1) | 898 (24.6) | 0.90 (0.78, 1.04) |
| p-interaction with age^b^ =1.00 | | | |  |  |  |  |

^a^Adjusted for age (continuous variable), sex, school education, body mass index, smoking status, alcohol consumption, red meat intake, history of colonoscopy, history of diabetes, family history of colorectal cancer, use of statins, use of non-steroidal anti-inflammatory drugs, and polygenic risk score (continuous variable).

^b^Tested by additionally including a cross-product term of age (binary variable) and physical activity (categorical variable) along with the main effect terms in regression models.

**Abbreviation:** CI, confidence interval; CRC, colorectal cancer; LTPA, leisure time physical activity; OR, odds ratio; PA, physical activity; Q, quartile; Ref., reference.

**Supplementary Table S4**. Association of physical activity and colorectal cancer risk by polygenic risk score levels

| **PA/**  **LTPA** | **Low PRS^a^** | | | | **Medium PRS^a^** | | | | **High PRS^a^** | | | | **p- /q- value^d^** |
| --- | --- | --- | --- | --- | --- | --- | --- | --- | --- | --- | --- | --- | --- |
|  | **Case, N(%)** | **Control, N(%)** | **OR (95% CI)** | | **Case, N(%)** | **Control, N(%)** | **OR (95% CI)** | | **Case, N(%)** | **Control, N(%)** | **OR (95% CI)** | |  |
|  |  |  | **Model 1^b^** | **Model 2^c^** |  |  | **Model 1^b^** | **Model 2^c^** |  |  | **Model 1^b^** | **Model 2^c^** |  |
| Average lifetime PA | | | | |  |  |  |  |  |  |  |  | 0.52/0.57 |
| Q1 | 234 (22.6) | 349 (25.8) | Ref. | Ref. | 374 (23.6) | 323 (23.9) | Ref. | Ref. | 516 (22.2) | 345 (25.6) | Ref. | Ref. |  |
| Q2 | 250 (24.2) | 338 (25.0) | 1.10 (0.87,1.39) | 1.00 (0.78, 1.30) | 367 (23.2) | 337 (24.9) | 0.94 (0.76, 1.16) | 0.87 (0.69, 1.10) | 595 (25.6) | 339 (25.1) | 1.16 (0.96, 1.41) | 1.11 (0.90, 1.38) |  |
| Q3 | 249 (24.1) | 307 (22.7) | 1.21 (0.95, 1.53) | 1.14 (0.88, 1.48) | 413 (26.1) | 368 (27.2) | 0.96 (0.78, 1.18) | 0.91 (0.72, 1.14) | 548 (23.6) | 336 (24.9) | 1.08 (0.89, 1.31) | 0.99 (0.80, 1.23) |  |
| Q4 | 302 (29.2) | 359 (26.5) | 1.26 (1.00, 1.58) | 0.97 (0.75, 1.26) | 429 (27.1) | 326 (24.1) | 1.13 (0.92, 1.39) | 0.90 (0.71, 1.15) | 664 (28.6) | 329 (24.4) | 1.36 (1.12, 1.64) | 1.06 (0.85, 1.32) |  |
|  |  |  |  |  |  |  |  |  |  |  |  |  |  |
| Average lifetime LTPA | | | | |  |  |  |  |  |  |  |  | 0.046/0.18 |
| Q1 | 310 (30.0) | 344 (25.4) | Ref. | Ref. | 450 (28.4) | 326 (24.1) | Ref. | Ref. | 667 (28.7) | 344 (25.5) | Ref. | Ref. |  |
| Q2 | 277 (26.8) | 311 (23.0) | 0.99 (0.79, 1.24) | 1.11 (0.87, 1.41) | 395 (25.0) | 345 (25.5) | 0.83 (0.68, 1.02) | 0.87 (0.70, 1.08) | 572 (24.6) | 356 (26.4) | 0.82 (0.68, 0.99) | 0.85 (0.69, 1.04) |  |
| Q3 | 231 (22.3) | 360 (26.6) | 0.71 (0.57, 0.89) | 0.79 (0.62, 1.01) | 372 (23.5) | 341 (25.2) | 0.78 (0.64, 0.96) | 0.87 (0.70, 1.09) | 568 (24.5) | 313 (23.2) | 0.93 (0.77, 1.13) | 1.03 (0.83, 1.26) |  |
| Q4 | 217 (21.0) | 338 (25.0) | 0.71 (0.57, 0.90) | 0.81 (0.63, 1.04) | 366 (23.1) | 342 (25.3) | 0.76 (0.62, 0.94) | 0.91 (0.73, 1.14) | 516 (22.2) | 336 (24.9) | 0.79 (0.65, 0.96) | 0.88 (0.72, 1.09) |  |
|  |  |  |  |  |  |  |  |  |  |  |  |  |  |
| Latest PA | | | | |  |  |  |  |  |  |  |  | 0.30/0.57 |
| Q1 | 256 (24.7) | 350 (25.9) | Ref. | Ref. | 409 (25.8) | 309 (22.8) | Ref. | Ref. | 583 (25.1) | 357 (26.5) | Ref. | Ref. |  |
| Q2 | 266 (25.7) | 332 (24.5) | 1.09 (0.87, 1.38) | 1.19 (0.93, 1.53) | 405 (25.6) | 342 (25.3) | 0.88 (0.72, 1.08) | 0.93 (0.74, 1.16) | 608 (26.2) | 339 (25.1) | 1.09 (0.90, 1.31) | 1.16 (0.94, 1.42) |  |
| Q3 | 262 (25.3) | 328 (24.2) | 1.10 (0.87, 1.38) | 1.16 (0.90, 1.49) | 361 (22.8) | 354 (26.1) | 0.73 (0.59, 0.91) | 0.84 (0.66, 1.06) | 526 (22.6) | 332 (24.6) | 0.95 (0.78, 1.15) | 1.04 (0.84, 1.29) |  |
| Q4 | 251 (24.3) | 343 (25.4) | 1.01 (0.79, 1.29) | 1.04 (0.80, 1.36) | 408 (25.8) | 349 (25.8) | 0.81 (0.65, 1.01) | 0.86 (0.67, 1.09) | 606 (26.1) | 321 (23.8) | 1.13 (0.92, 1.38) | 1.20 (0.97, 1.50) |  |
|  |  |  |  |  |  |  |  |  |  |  |  |  |  |
| Latest LTPA | | | | |  |  |  |  |  |  |  |  | 0.57/0.57 |
| Q1 | 319 (30.8) | 349 (25.8) | Ref. | Ref. | 473 (29.9) | 328 (24.2) | Ref. | Ref. | 678 (29.2) | 358 (26.5) | Ref. | Ref. |  |
| Q2 | 300 (29.0) | 342 (25.3) | 0.96 (0.77, 1.19) | 1.04 (0.82, 1.32) | 436 (27.5) | 333 (24.6) | 0.88 (0.72, 1.08) | 0.92 (0.74, 1.15) | 667 (28.7) | 336 (24.9) | 1.03 (0.86, 1.24) | 1.10 (0.90, 1.34) |  |
| Q3 | 195 (18.8) | 305 (22.5) | 0.69 (0.53, 0.88) | 0.80 (0.61, 1.04) | 345 (21.8) | 365 (27.0) | 0.62 (0.50, 0.76) | 0.73 (0.58, 0.91) | 497 (21.4) | 328 (24.3) | 0.78 (0.65, 0.95) | 0.94 (0.76, 1.16) |  |
| Q4 | 221 (21.4) | 357 (26.4) | 0.67 (0.53, 0.85) | 0.85 (0.66, 1.09) | 329 (20.8) | 328 (24.2) | 0.66 (0.53, 0.81) | 0.86 (0.68, 1.09) | 481 (20.7) | 327 (24.2) | 0.76 (0.62, 0.92) | 0.92 (0.74, 1.14) |  |

^a^PRS was categorized into low, medium, and high levels using tertiles of PRS among controls.

^b^Adjusted for age and sex.

^c^Additionally adjusted for school education, body mass index, smoking status, alcohol consumption, red meat intake, history of colonoscopy, history of diabetes, family history of colorectal cancer, use of statins, and use of non-steroidal anti-inflammatory drugs.

^d^Tested by additionally including a cross-term of PRS (categorical variable) and physical activity (categorical variable) in model 2; The q-values are the FDR-adjusted p values.

**Abbreviation:** CI, confidence interval; LTPA, leisure time physical activity; OR, odds ratio; PA, physical activity; PRS, polygenic risk score; Q, quartile; Ref., reference.


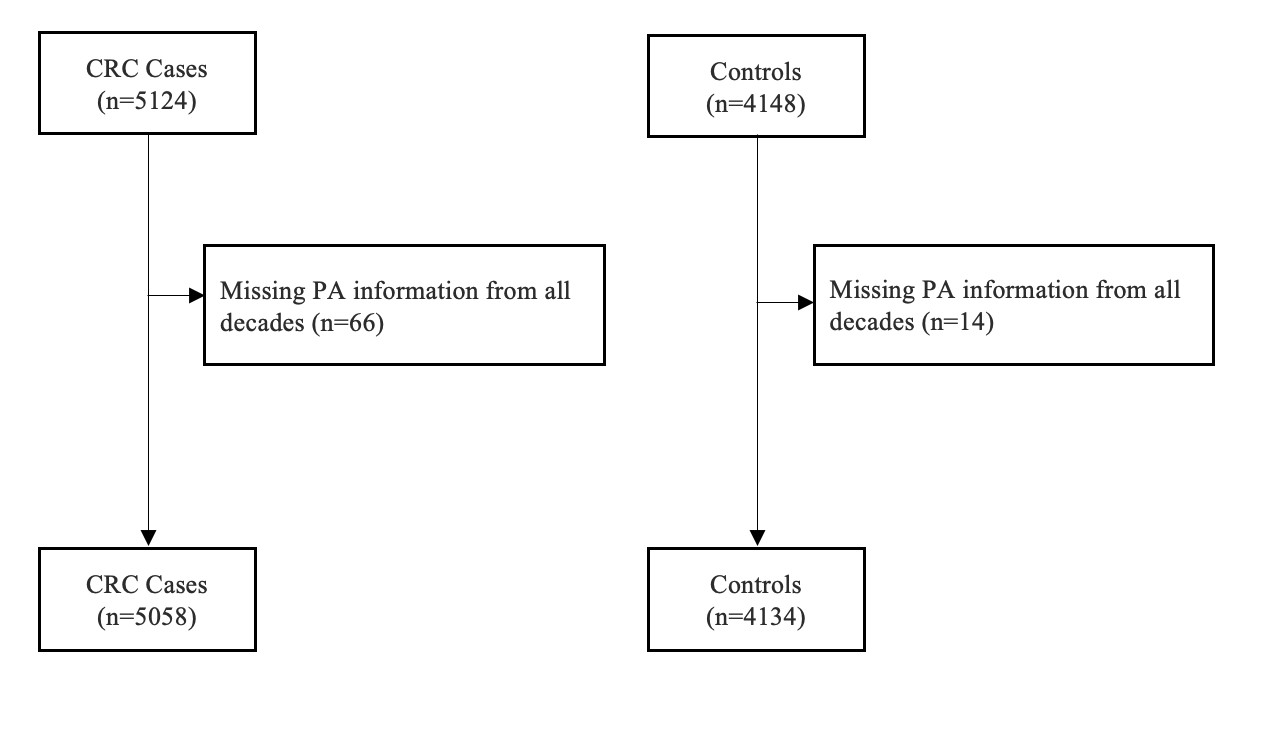


**Supplementary Figure S1.** Flowchart of inclusion of study participants.

**Abbreviation:** CRC, colorectal cancer; PA, physical activity.
